# A functional pipeline framework for landmark identification on 3D surface extracted from volumetric data

**DOI:** 10.1371/journal.pone.0187558

**Published:** 2017-11-09

**Authors:** Pan Zheng, Bahari Belaton, Iman Yi Liao, Zainul Ahmad Rajion

**Affiliations:** 1 Faculty of Engineering, Computing and Science, Swinburne University of Technology Sarawak Campus, Kuching, Malaysia; 2 School of Computer Sciences, Universiti Sains Malaysia, Penang, Malaysia; 3 School of Computer Science, The University of Nottingham Malaysia Campus, Semenyih, Malaysia; 4 School of Dental Sciences, Universiti Sains Malaysia, Kubang Kerian, Malaysia; 5 College of Dentistry, King Saud bin Abdulaziz University for Health Sciences, Riyadh, Kingdom of Saudi Arabia; Xiamen University, CHINA

## Abstract

Landmarks, also known as feature points, are one of the important geometry primitives that describe the predominant characteristics of a surface. In this study we proposed a self-contained framework to generate landmarks on surfaces extracted from volumetric data. The framework is designed to be a three-fold pipeline structure. The pipeline comprises three phases which are surface construction, crest line extraction and landmark identification. With input as a volumetric data and output as landmarks, the pipeline takes in 3D raw data and produces a 0D geometry feature. In each phase we investigate existing methods, extend and tailor the methods to fit the pipeline design. The pipeline is designed to be functional as it is modularised to have a dedicated function in each phase. We extended the implicit surface polygonizer for surface construction in first phase, developed an alternative way to compute the gradient of maximal curvature for crest line extraction in second phase and finally we combine curvature information and K-means clustering method to identify the landmarks in the third phase. The implementations are firstly carried on a controlled environment, i.e. synthetic data, for proof of concept. Then the method is tested on a small scale data set and subsequently on huge data set. Issues and justifications are addressed accordingly for each phase.

## Introduction

On a 3D surface, landmarks are geometrical features used as salient points of origin in locating other features or as points from which measurements can be taken. They are usually easier to create and much less expensive to render than other types of geometry primitives, i.e. lines and surfaces, and efficiently represent saliency of features of an object. For either 2D or 3D image data, a landmark can be in forms of a point, a line, a surface and even a sub-volume [1]. This study focuses on the landmarks in the form of points.

In computer graphics, data visualisation, image processing and related fields, landmarks are used to facilitate various processes and applications. One may find landmarks to be useful in registration [2], matching [3], segmentation [4], reconstruction [5], recognition [6], measurement [7] and motion analysis [8]. A usual and common practice of landmark identification is to conduct the landmarks placement manually. It may not serve those processes efficiently because it suffers from drawbacks such as tedious, labour-intensive, time-consuming and error-prone.

Meanwhile, with the advancement of 3D scan modalities, i.e. computerised tomography, magnetic resonance imaging, ultrasound and so on, large amount of volumetric data are available for scientific research and analysis. The data can be defined as a 3D array where every location in 3D array has an intensity associated with it. It can also be considered as a 3D grid where in each of the vertices and crossing of the grid it has a scalar value assigned. Volumetric data is usually formed by stacking up a series of 2D image data. There are often two ways to study and analyse 3D volumetric data. One way is to study the 2D images comprised the data, perform tasks such as feature extraction, segmentation and then revert to 3D volumetric data. Another way is to perform surface extraction based on the intensity (isovalue) of the volumetric data and carry out necessary tasks on the surface (isosurface). This study follows the latter way.

The primary objective of this study is to attempt to automate the landmark, i.e. feature point, identification on 3D surfaces. We propose a functional pipeline framework which takes volumetric data as initial input and produce output as landmarks on the surface extracted from the volumetric data. The pipeline approach breaks down a task and modularised into several subtasks and sequentially solves the subtasks. Functional is defined as each phase of the pipeline has a dedicated function of its own. The pipeline proposed is consist of three phases, which are surface construction, feature line extraction and landmark identification respectively. In the first phase of surface construction, we study various surface construction methods and find an efficient algorithm to extend to fit into the pipeline. The second phase is about feature line extraction. In this phase, the feature lines considered are crest lines. Various geometry components and properties associated with crest line extraction are investigated. The last phase focuses on feature point identification on 3D curves.

## Related work

There have been intensive researches and studies conducted on landmarks identification in domains such as computer graphics and image processing as well as in practical fields, i.e., biology and medical image diagnosis. In practical fields, the landmarks are initially identified manually [9] with various assisting equipment and tools. Subsequently with the development of different kinds of scan modalities, the landmarks can be identified by software with human intervention [10, 11]. In the last two decades, endeavours of automatic means of landmarks identification draw more and more efforts.

Majority of the works of 3D landmark identification use geometry approaches. One of the early studies is reported by Thirion [12]. The feature points are defined as extremal points. The extremal points are calculated with information of curvatures and extremality coefficient. The study then uses the feature points as a tool to do 3D registration. It is a pure differential geometry approach to obtain the landmarks. The work is later extended in [13].

Landmark can be used in the process of registration. It is also possible to be the other way round. Conde [14] presents a method of 3D face feature points extraction based on Spin Images registration. SPIN Images are computed for face models. The Support Vector Machine (SVM) is used to classify the correspondence of Spin Image to feature points. The method is implemented on 3D range mesh. The feature points detected are nose tip point and inner corner points of both eyes. Curvature information plays a vital role as well. One of the notable points of this study is that it introduces Machine Learning technique, i.e. SVM, to 3D geometry analysis.

Landmark identification can also be formulated as a probabilistic inference problem. Azouz [15] proposed a method using probabilistic graphical model, i.e., Markov Random field (MRF). The landmarks are identified over a whole human body model and connected in pairwise to form an undirected graph. The method consists of two major steps, which are learning and inference. The learning process is achieved by identifying the parameters of the nodes and the edges of MRF. The inference over MRF is to find values of the random variables maximizing the joint probability. The method introduces techniques in graph theory and machine learning to solve the problem.

Steder [16] proposed a normal aligned radial feature (NARF) method to extract point features on 3D range data. The method first combines information of 2D image and 3D range data to find the boundaries of a 3D scene. Point features are located with neighbour score and dominant directions.

Applying machine learning techniques on 3D point features and landmark identification becomes more and more popular in recent years. Cresusot reports a Linear Discriminant Analysis (LDA) approach to solve this problem [17]. The method calculates shape descriptor values of a template model (mean mesh) of a human face scans, uses probability density function (PDF) to compute the shape description scores from shape descriptor values and finally employs LDA to form landmark score function (detector function) to discover the landmarks on a test face. The advantage of using machine learning approach is that applying the model to extract features is more efficient than carrying out complex geometrical analysis of different given 3D objects. A more comprehensive explanation and an extension of the work are reported in [18].

Chen [19] presents a statistic method to extract landmark features. The landmark features are defined as “Schelling points”, which essentially means the mesh points have saliency in semantics. The research is conducted first by online surveying. 3D shapes are put online and people (Internet users) are paid to choose the most salient points on the 3D meshes. After the survey, consistency, distribution, symmetry and some behaviour analyses are conducted on the survey data. Then a regression model is developed based on these data. Finally they proposed a prediction model based on the statistic model for Schelling point extraction. This study is refreshing. It tackles the problem in a different angle with some means usually found in social science.

Another approach is using Extended Gaussian Image with Low-density Bins (ELB) [20]. This method partitions 3D mesh surfaces (human faces) into several patches. ELB histogram is calculated for each patch. Concatenation of all the histogram of patches forms Histogram of Shape Normal Information (HoSNI) which is used as surface descriptor. The HoSNI of landmarks are calculated in face models and clustered with KMeans Clustering. The centroids of the clusters are used as template of the landmarks. For a given testing face, HoSNI is computed and compared with the template with chi-square test to identify the landmarks.

As machine learning techniques are prevalent in solving various problems, one of the latest feature/key point method [21] treats the problem as a binary classification problem, where a 3D descriptor-specific detector plays an important role to identify the points. The descriptor is obtained by a training process and then the method is tuned by Random Forest.

Some recent 2D feature point detection uses RGB colour gradient and colour component statistics model [22]. AdaBoost and improved Active Appearance Model(AAM) are used in face feature points detection [23]. A neural network approach for 3D feature point exaction is presented in [24] and used in accurate positioning of vehicles.

Current literatures cover a wide spectrum of techniques, data and applications, each of which has its own uniqueness. Nevertheless, there are some issues which are worth noting and discussion.

- Data. We notice that majority of these methods use 3D data in the format of range data, which essentially are mesh formed by sampling points on the surface of an object. Very few methods work on 3D data in volume and employ volume information, e.g. voxel, into the solution.
- Design of the method. Model and template based approaches have quite influences on all methods. There are some limitations of this kind of method [25]. The model construction is a time consuming task that requires either extensive statistical analysis or delicate machine learning algorithms. It could be insufficient for real-time and interactive systems and the design is restricted for specific off-line applications. The issues of model based approach are still contentious.
- Applications and Reusability. Some methods focus on scenes and human faces generated by 3D range scans. The methods are generally application-oriented and lack of cross-application ability.

Enlightened by previous efforts, we propose a conceptual framework and method for landmarks identification which bears the following characteristics.

- Modularise: The proposed framework is modularised. The problem is divided and conquered into subtasks.
- Geometry based: It is more intuitive to solve a geometry problem with geometry methods.
- Geometric invariance: Landmarks are not affected by rigid transformation.
- Volume information: We would like to see how volume information impacts the landmarks identification on a 3D surface.
- Reproducibility and reusability: The method should not be only used for specific context but possible to reuse for other scenarios and applications.

## Design of the framework

**Fig 1:**
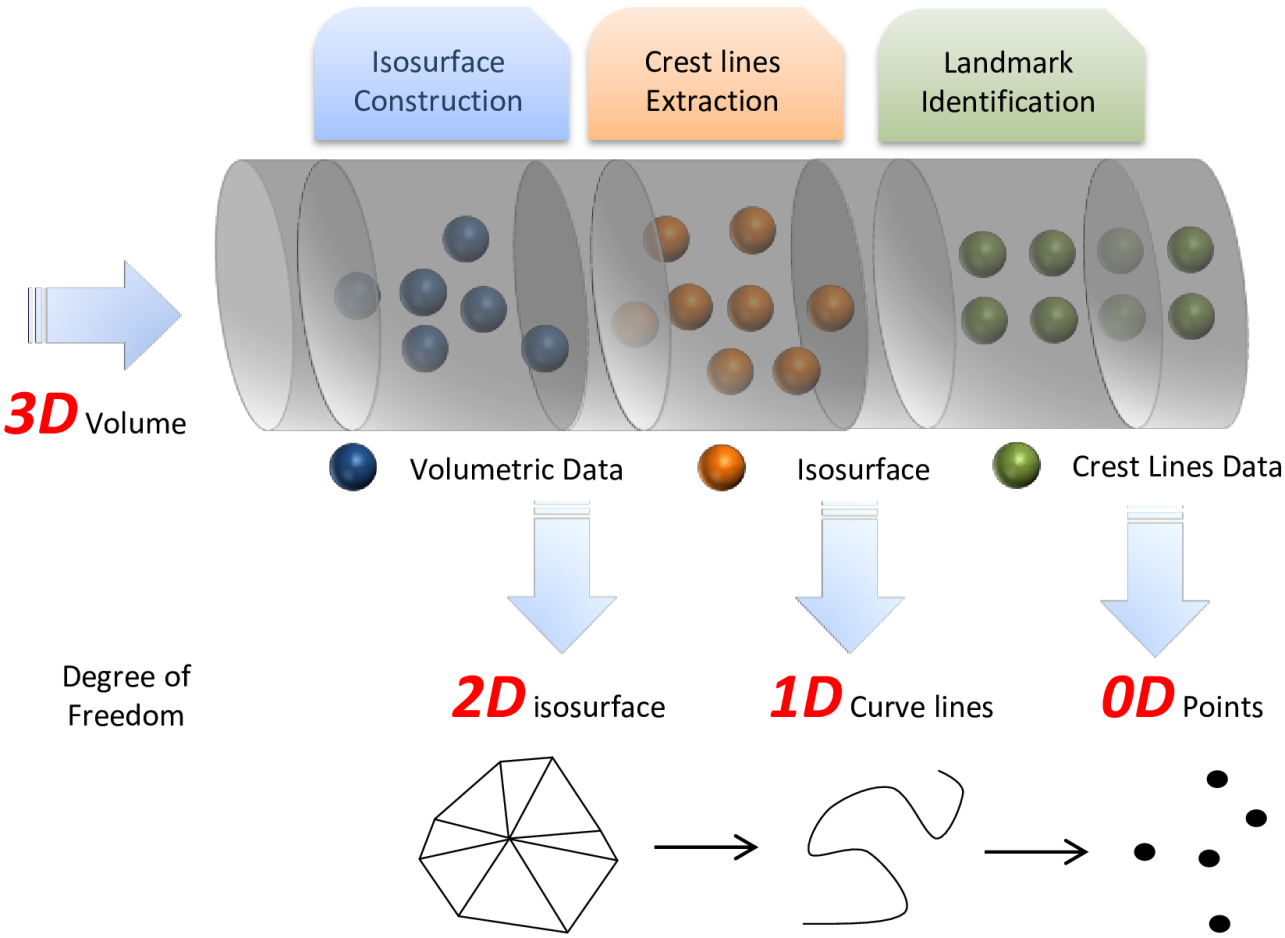
The conceptual illustration of the functional pipeline.

The general method of identifying landmarks consists of two steps, which are detection and localisation [26]. In the detection stage, the main task is to determine the geometrical properties of the landmark. In the localization stage, the coordinates of the landmarks are estimated. There is no distinctive division between these two steps. It provides a basic idea of landmark identification. Our aim is to identify the landmarks on a surface extract from a volumetric data. Landmark points are geometry primitives with 0D degree of freedom whereas volumetric data is 3D degree of freedom. Naturally the problem can be viewed as a dimension reduction process. A high level architectural design is shown in Fig 1. It illustrates the overall design of this study.

The pipeline is designed to be functional as it is modularized to have a stand-alone function for each phase. A dedicated task is assigned to each stage and there is no inter-phase interference. The output of each phase has high potential of reusability. The input of the pipeline is pre-processed 3D volume data which is in ASCII format and can be stored in a 3D array. The first phase is surface extraction. In this phase we use implicit surface polygonizer [27]. The second phase is crest lines extraction. We implement the method addressed by [12, 28]. The third phase is landmark identification on the feature lines extracted on the surface. We propose a method of landmark selection based on the curvedness of the crest points. All the existing methods are extended and tailored to fit the pipeline design. The following sections elaborate each phase in detail.

## Surface construction

**Fig 2:**
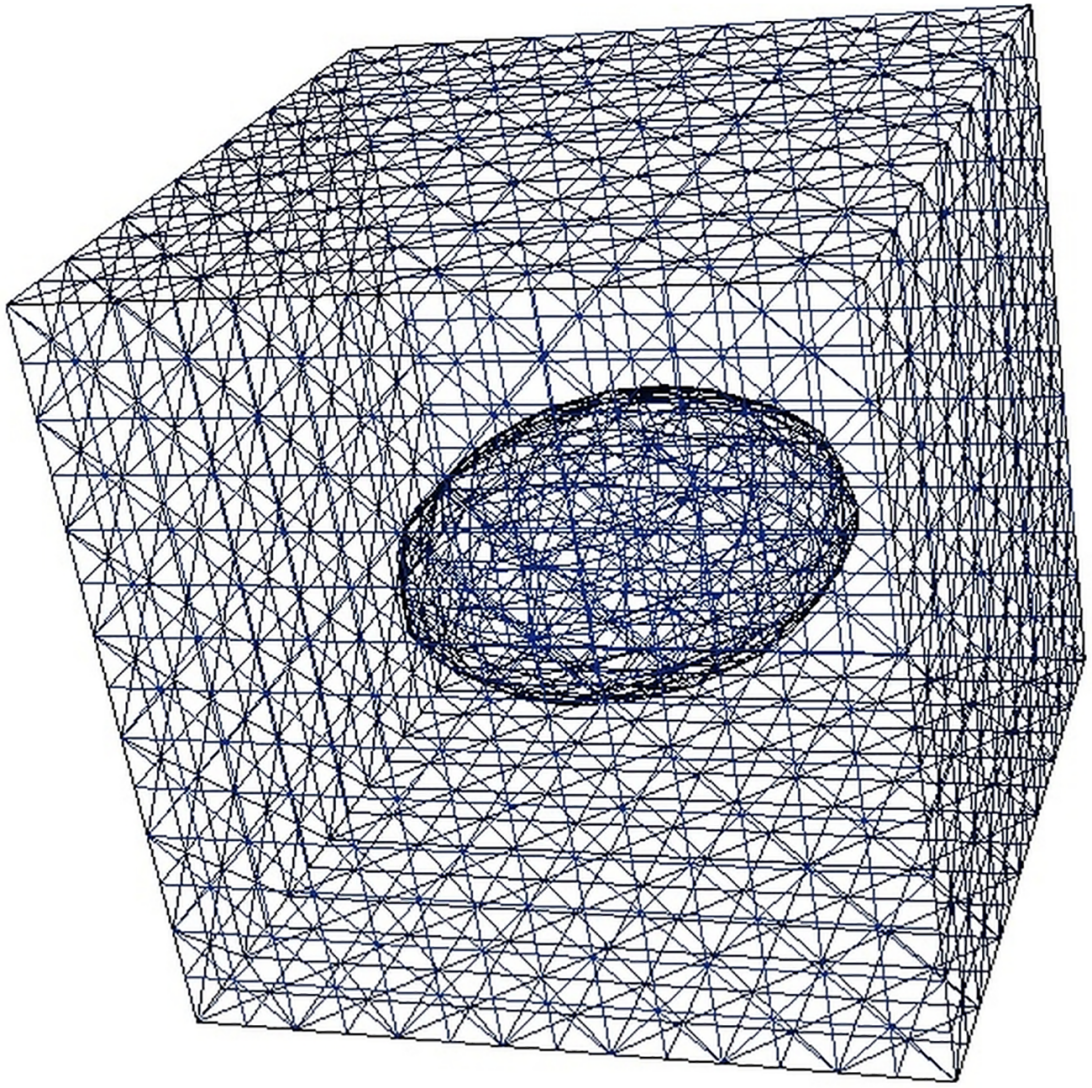
An example of isosurface (inner ellipsoid object) extracted from a 3D volumetric data (bounded by cube).

In this study the 3D volume is pictured as a cuboid, i.e. a right parallelepiped, bound by six rectangular faces. A set of discrete-contiguous scalar values are defined within. Thus the volume data can be defined as *p* = *v*(*x*, *y*, *z*), where v is the function of volume; *x*, *y* and *z* are the coordinates of the sampled points in the volume; *p* is the intensity value (scalar) of the corresponding points at the coordinate. The scalar values can present values of pressure, temperature and density. They are uniformly sampled in a structured grid. To investigate the values dissemination in the volume, a constant scalar value *p*_*c*_ may be defined. All the points that have same value as *p*_*c*_ can be found inside the volume. These points can be determined by *p* − *p*_*c*_ = 0, which is an implicitly defined 3D surface. This kind of surface is usually called an isosurface that contains the locales of all points that have constant value *p*_*c*_(Fig 2).

### Implicit surface polygonization

As the application of implicit formulations has been intensively studied in the field of geometry, possible solutions for surface extraction have been investigated. One of these results is the implicit surface polygonisation which was described by Jules Bloomenthal [27]. With reference to his previous works, this research studies the method of isosurface extraction from another perspective, rather than conventional methods, i.e. Marching Cubes [29] and its variants. The basic idea of implicit surface polygonizer is described in following,

- A starting point is initiated randomly in the volume.
- From the starting point, propagation is conducted with step-size of fraction of a cube in reasonable iterations. With reference to the surface function, this is to find a point with opposite sign. The sign is computed by comparison of the iso-value and the point values.
- Binary subdivision is applied to approximate a seed point. The seed point should be the first point found on the surface.
- An initial cube is created at the point.
- Then, piecewise-linear method along with the surface function is used to do the evaluation on the neighbouring cubes for surface intersection.
- Eventually, all cubes that contain the surface will be discovered.
- Within each cube, linear interpolation produces the vertices of triangle/polygon patches of the surface.

**Fig 3:**
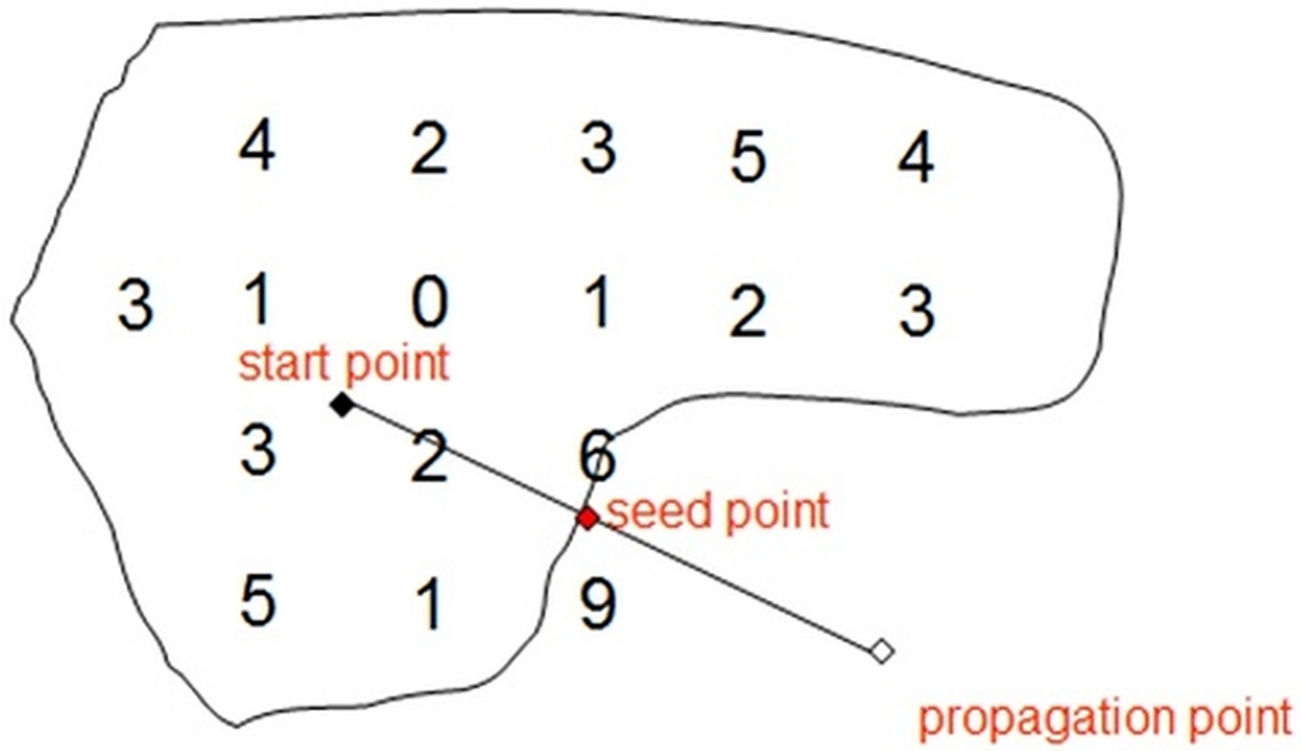
A propagation point is found with negative sign to starting point.

Above scenario can be elaborated in a 2D context for a clear illustration. Refer Fig 3, the threshold value is 6 in this case. The starting point, denoted with solid point, is positive. The propagation point, denoted in hollow point, is negative

**Fig 4:**
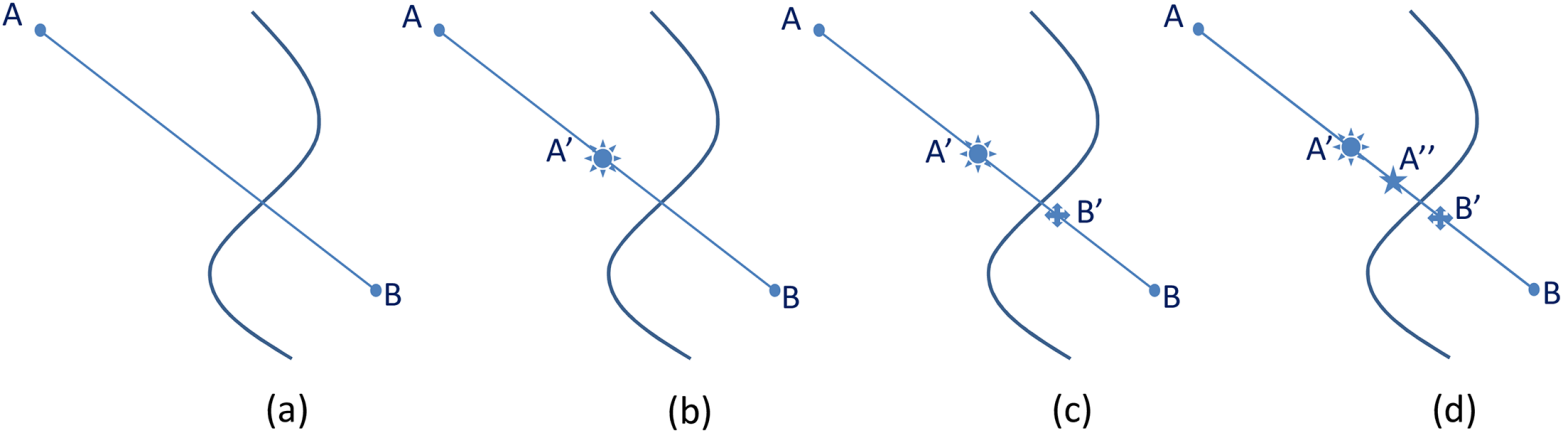
Binary subdivision.

Binary subdivision is a delicate initial approximation method to find the seed point, shown in Fig 4.

**Fig 5:**
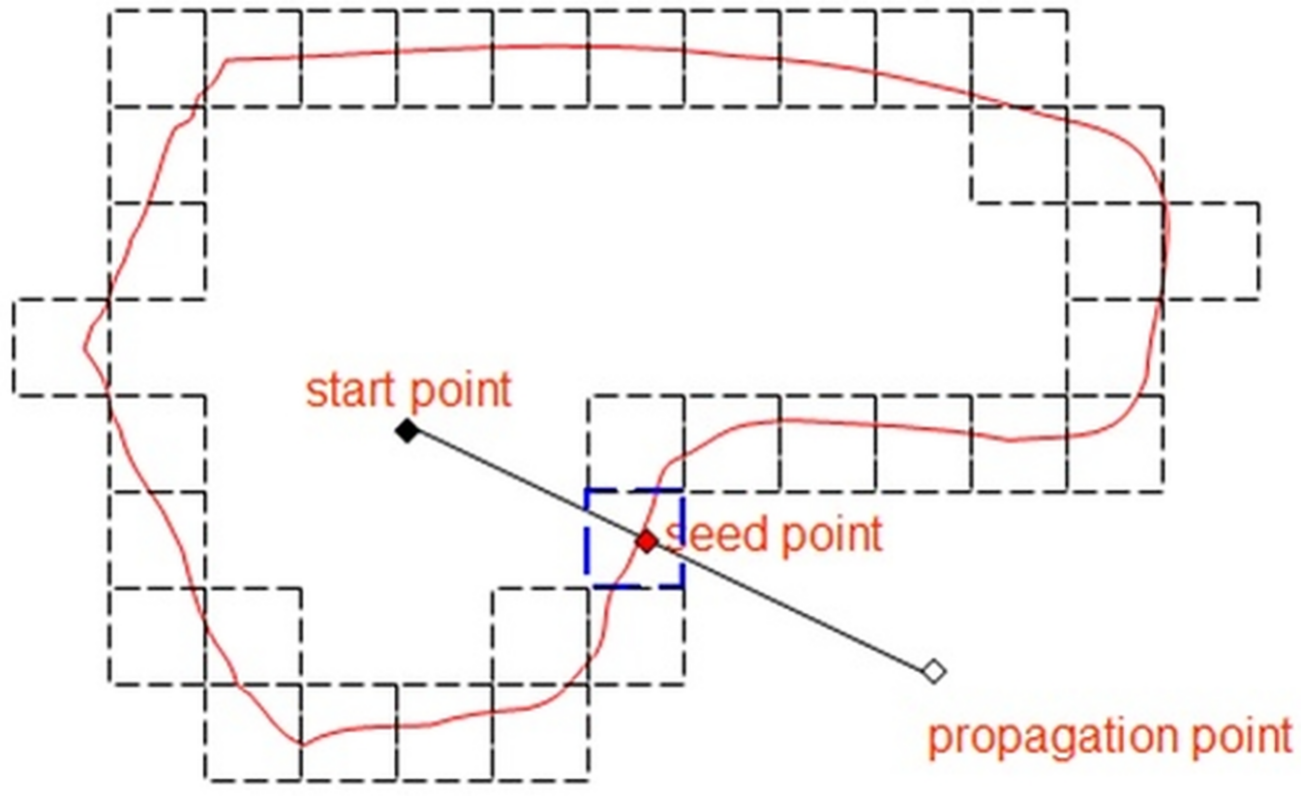
Contour grid generation with piecewise-linear method.

As shown in Fig 4, we have {*P_a_*: *A*, *A*′, *A*′′…} ⊂ ℜ^+^ and {*P_b_*: *B*, *B*′ …} ⊂ ℜ^−^. Initially a point belongs to *P*_*a*_ or *P*_*b*_ which is decided by the sign of the result of point value minus isovalue. In this case, A and B have different sign, i.e. polarity. Then there is apparently a zero value which is the isosurface (Fig 4(a)) in-between. A middle point is taken in between A and B (Fig 4(b)), and the sign is evaluated. If the sign is positive, A’ in this case, a middle point is taken in between A’ and B (Fig 4(c)). the middle point of A’ and B is negative, i.e. B’. subsequently a middle point is taken between A’ and B’, which is positive, i.e. A” (Fig 4(d)). Depending on the sign of the middle points, this process can continue iteratively. Eventually a point that extremely approximates the surface can be converged. And that very point is the seed point to start the surface track traversal. Fig 5 below shows the square propagation of the polynigozer after the seed point placement and a series of squares containing the iso-line are obtained.

**Fig 6:**
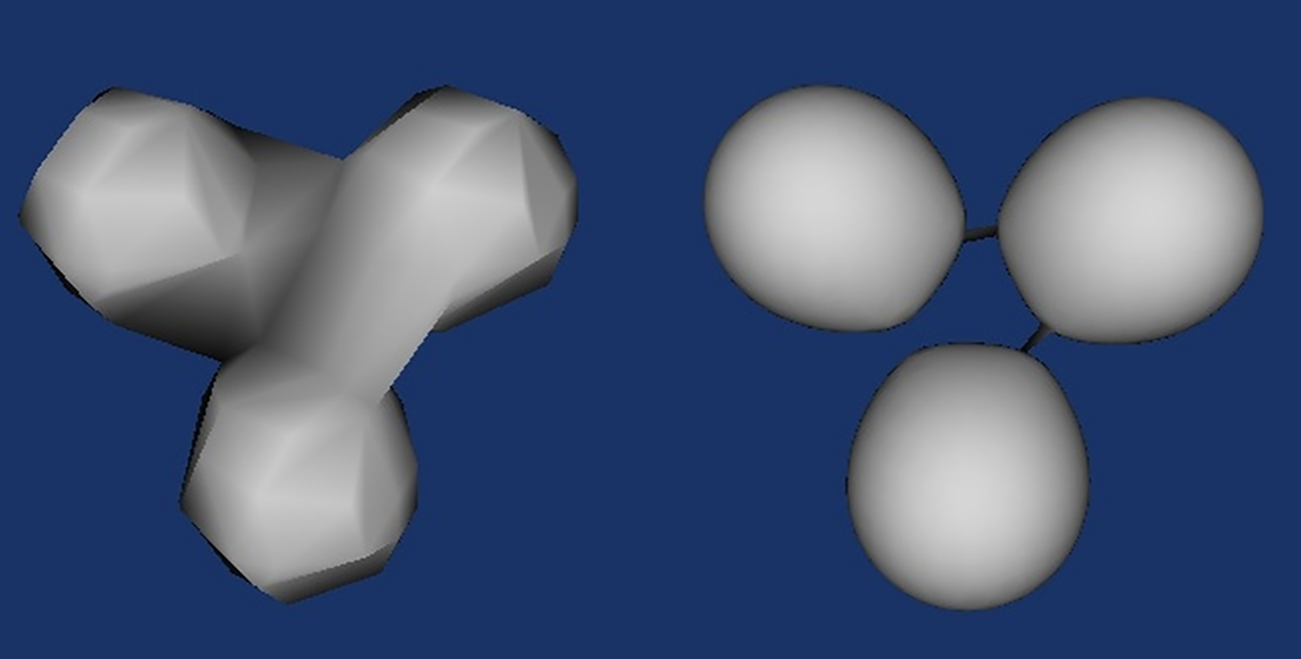
The cube size problem of implicit surface polygonizer.

One major advantage of implicit surface polygonizer over other surface extraction methods is the time complexity. Exhaustive methods usually have a complexity of *O*(*n*^3^) whereas the implicit surface polygonizer using tracking partition has a complexity of about *O*(*n*^2^). The disadvantage of it can be the decision of the cube size, where too big the cube size is, detail of the object may lose; too small the cube size it, the object may be separated (Fig 6). One solution of the cube size issue is to make the cube size proportional to the data size. Depending on the experiment needs the cube size may be thousandth or 10 thousandth of the data size rather than a fix cube size.

### An extension of implicit surface polygonizer

The basic idea is that the function reads the volume data in a three-dimension array. For each coordinate passed in, the function does a tri-linear interpolation against the scalar values of the four vertices of the containing cube/voxel to determine the returning value (Table 1).

**Table 1:** The pseudocode of the extension of previous implicit surface polygonizer.

**Data:** scalar value of points in discrete volume data	
**Result:** evaluation results of the implicit function	
**Function:** ImplicitSurFunc(px, py, pz)	
01	int flr_x, flr_y, flr_z
02	float fracx, fracy, fracz
03	float v000,v001,v010,v011,v100,v101,v110,v111
04	float v00,v01,v10,v11,v0,v1
05	float vvv;
06	flr_x≔ abs(floor(px))
07	flr_y≔ abs(floor(py))
08	flr_z≔ abs(floor(pz));
09	fracx≔ px—(int)px
10	fracy≔ py—(int)py
11	fracz≔ pz—(int)pz;
12	v000≔ data[flr_x][flr_y][flr_z]
13	v001≔ data[flr_x][flr_y][flr_z+1]
14	v010≔ data[flr_x][flr_y+1][flr_z]
15	v011≔ data[flr_x][flr_y+1][flr_z+1]
16	v100≔ data[flr_x+1][flr_y][flr_z]
17	v101≔ data[flr_x+1][flr_y][flr_z+1]
18	v110≔ data[flr_x+1][flr_y+1][flr_z]
19	v111≔ data[flr_x+1][flr_y+1][flr_z+1];
20	v00 = v000+fracz*(v001-v000)
21	v01 = v010+fracz*(v011-v010)
22	v10 = v100+fracz*(v101-v100)
23	v11 = v110+fracz*(v111-v110);
24	v0 = v00+fracy*(v01-v00)
25	v1 = v10+fracy*(v11-v10);
26	v = v0+v1;
	/* or we can have one line as
27	vvv≔ v000*(1-fracx)*(1-fracy)*(1-fracz)+ v100*fracx*(1-fracy)*(1-fracz)+ v010*(1-fracx)*fracy*(1-fracz)+ v001*(1-fracx)*(1-fracy)*fracz+ v110*fracx*fracy*(1-fracz)+ v101*fracx*(1-fracy)*fracz+ v011*(1-fracx)*fracy*fracz+ v111*fracx*fracy*fracz;*/
28	return vvv

**Fig 7:**
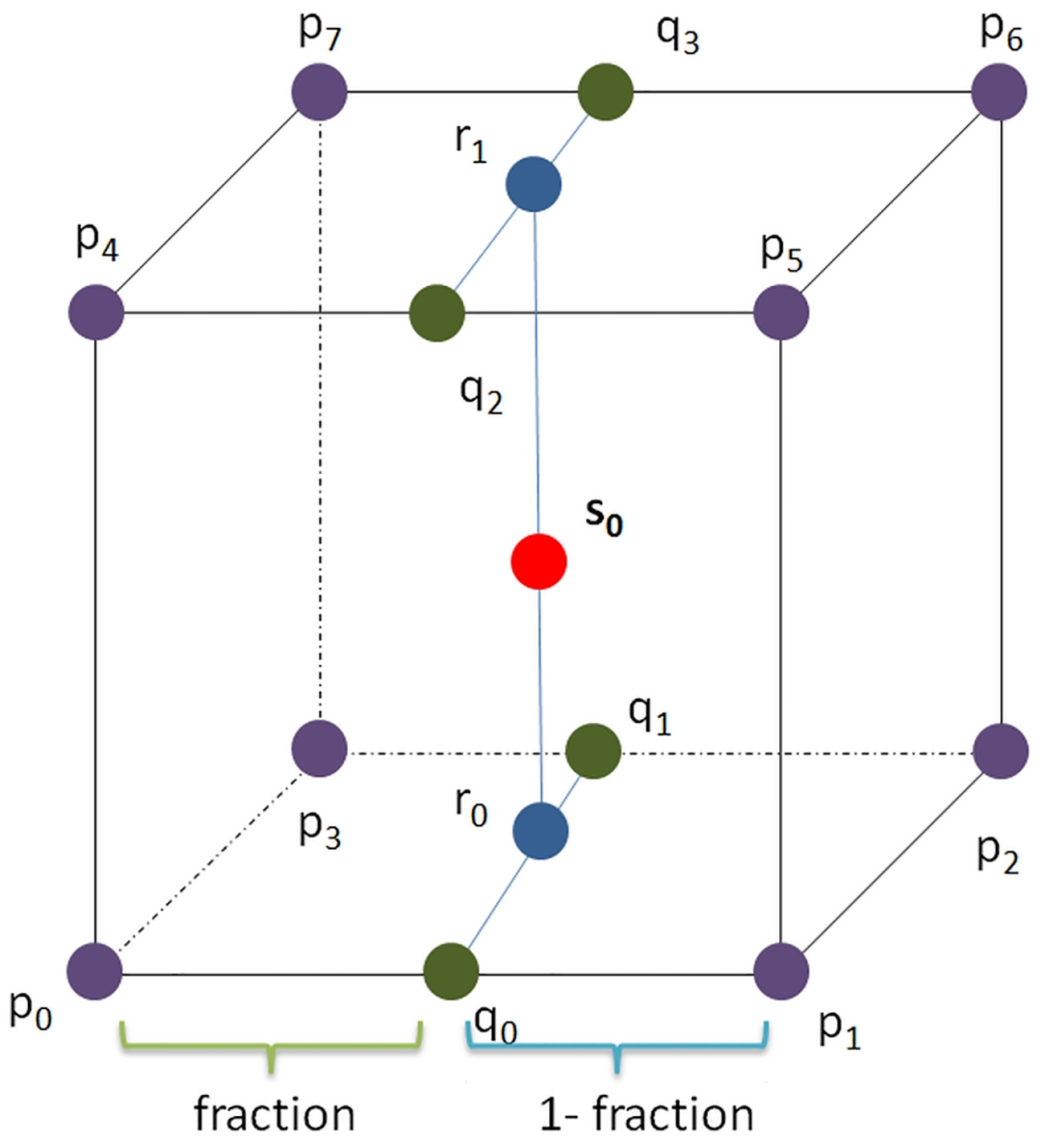
Tri-linear interpolation inside a cube/voxel.

In detail line 01 to 05 define the variable required for the function. Line 06 to 11 calculates the fractions for interpolation. Lines 12 to 19 are the scalar intensity of the 8 vertices in the respective cube/volex of the volume (highlighted in purple in Fig 7, i.e. *p*_*i*_). Line 20 to 23 implements the interpolation to obtain the values along the edges of the cube (highlighted in green in Fig 7, i.e. *q*_*i*_). Line 24 to 25 shows the interpolation to obtain intermediate values needed for final interpolation (highlighted in blue in Fig 7, i.e. *r*_*i*_). Line 26 shows the final interpolation to obtain the scalar intensity of respective vertex on the surface (highlighted in red in Fig 7, i.e. *s*_0_). Line 20 to line 26 may be summarised in line 27.

**Fig 8:**
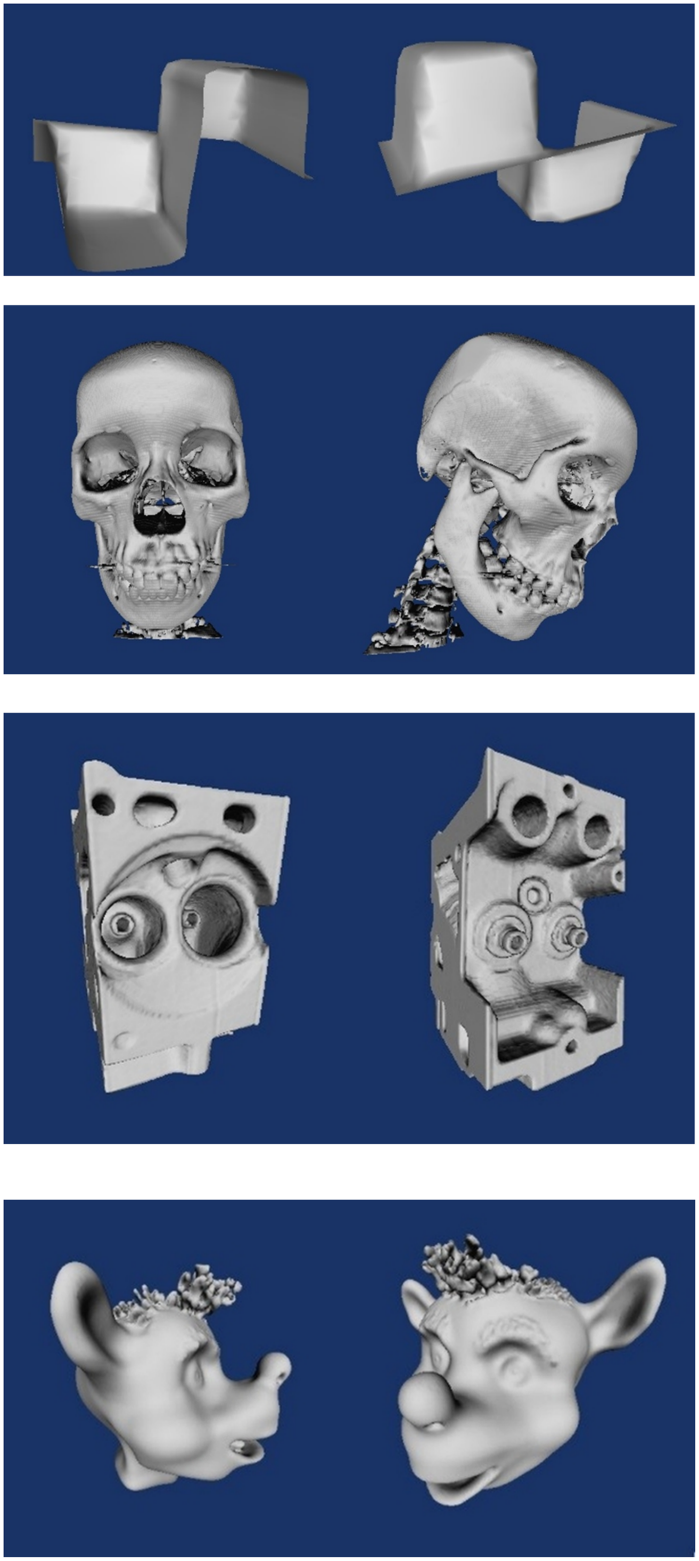
Surface construction results using extended implicit surface polygonizer. **(a)**Double glazing window temperature. **(b)**preprocessed craniofacial CT data. **(c)**Engine block data. **(d)**Animated bear.

Fig 8 shows the result of extended implicit surface polygonizer on generating surfaces on varoius 3D volumetric data (Table 2). The resolution of the results depends on the traversal cubes as well as the original resolutions of the volumetric data.

**Table 2:** Description of the data used in the experiment of surface extraction.

Data	Temperature	Craniofacial	Engine block	Animated Bear
Dimension (*x* × *y* × *z*)	10 × 10 × 18	256 × 256 × 196	256 × 256 × 110	256 × 256 × 256
Pixel depth	8 bit	16 bit	8 bit	8 bit
Voxel spacing (*x* × *y* × *z*)	1 × 1 × 1	0.47 × 0.47 × 1.25	1 × 1 × 1	1 × 1 × 1
Description	Discrete sampling of double glazing window temperature data.	A craniofacial CT scan of a female patient provided by Hospital USM.	A CT scan of two cylinders of an engine block from the VolPack volume rendering library by Stanford University.	It is an animated bear head data. It is obtained from one of the Andreas Bærentzen’s research paper [30] with his permission

For the efficiency comparisons, the same datasets are tested for surface constructions using well-known surface construction algorithm Marching Cubes [29].

Table 3 shows the quantitative comparison of the surface construction by using Marching Cubes and extended implicit surface polygonizer. For the four sets of data, the table compares the number of triangles and number of vertices generated by both methods. Except Craniofacial data, Marching Cubes generated smaller number of triangles and vertices, however the crucial part is the Ratio. The Ratio denotes the rate of triangles per vertex. The ratios of implicit surface polygonizer are higher than Marching Cubes method in all four data sets. It means that, for same amount triangles, implicit surface polygonizer tends to use less number of vertices. In other words, implicit surface polygonizer can use the vertices more efficiently than Marching Cubes.

**Table 3:** Comparison of Marching Cubes and extended implicit surface polygonizer.

		Marching Cubes	Extended Implicit surface polygonizer
Temperature	# of Triangles	1,026	1,176
	# of Vertices	556	632
	Ratio	1.845	1.861
Craniofaicial	# of Triangles	2,236,443	2,021,048
	# of Vertices	1,390,036	1,009,539
	Ratio	1.609	2.002
Engine block	# of Triangles	61,296	69,252
	# of Vertices	31,143	34,582
	Ratio	1.968	2.003
Animated bear	# of Triangles	110,539	113,816
	# of Vertices	55,277	56,902
	Ratio	1.999	2.000

One practical issue we encountered is the boundary problem. To avoid the tracking cubes process to propagate infinitely, boundary settings are necessary. The original implementation cannot be used to extract surface of a manifold-with-boundary object, although global bounds can be hard-coded to trim the surface based on the information given in the data, e.g. dimension. For a manifold-with-boundary object, points on the boundary curve need to be explicitly computed. One of such approach has been discussed in [4]. We constrain the surface propagation based on the dimension of the volumetric data. If the surface propagates outside the dimension, the program does not generate any geometry for the surface.

Another issue is the starting point placement for fast detection of a seed point. The starting point is controlled by the implementation. Since the Houndsfield value of CT data for different threshold values of human body are generally known, the location of the starting point can be supervised and approximated rather than using a random selection strategy.

## Feature line extraction

The second phase of the pipeline is feature line extraction. There are various types of feature lines, i.e. silhouettes, contour, hatching strokes and so on, which can be used to express, illustrate and describe objects, art form and even cartoon. The feature line that we are interested in is the crest line. Crest lines illustrate the ridges and ravines of a surface. Geometrically crest lines are produced by connecting crest points. Crest points are surface points that are calculated by zero-crossing the surface extremals. Surface extremals exhibit the variations of principal curvatures of surface vertices on their principal directions.

**Fig 9:**
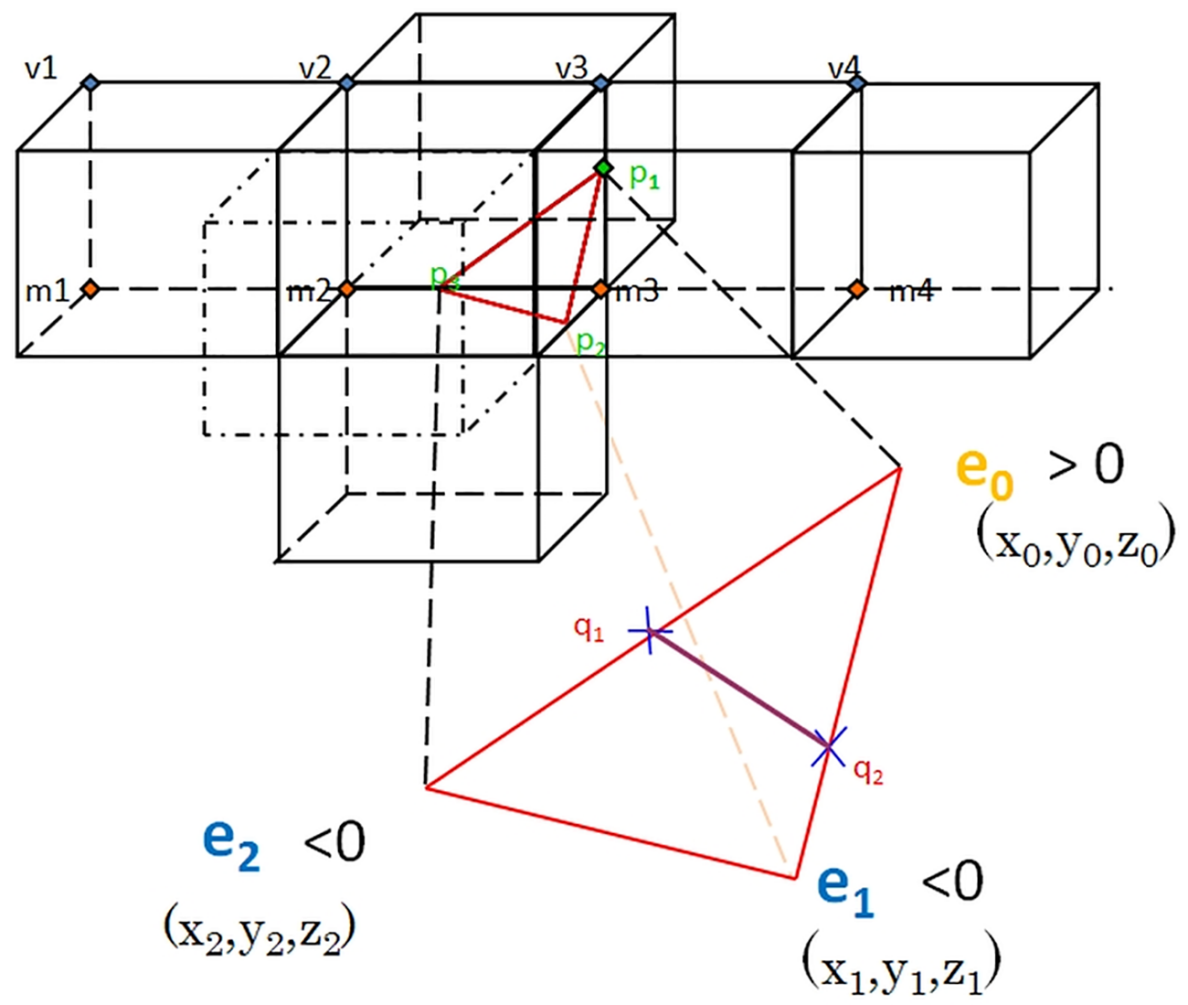
Crest lines calculation within a triangle facet of a surface.

Fig 9 illustrates the basic idea of crest line extraction. The first step is to calculate the extremal value of the all vertices of a surface. Then for each triangle patch of the surface, if the extremal values of all three vertices of triangle are with same sign, then there is no zero-crossing, therefore, no crest line in the triangle. If there is one vertex has different sign from the other two vertices, there are zero-crossings, hence, a crest line within the triangle. The triangle shown in Fig 9 has one vertex with the sign of the extremal value that is different from the other two vertices, i.e. *e*_0_ > 0 and *e*_1_, *e*_2_ < 0. Along the edge *e*_0_ − *e*_1_, there must be some point with the extremal value of zero, namely a zero-crossing. The coordinates of the zero-crossing can be calculated by linear interpolation. The next task is to calculate the extremal values of all vertices of the surface. We have the following equation,

$$\begin{array}{c} e_{max}=\vec{\nabla k_{max}}\cdot \vec{t_{max}} \end{array}$$ (1)

The value of *e* can be calculated by the dot-product of the gradient of maximal principal curvature and the corresponding principal direction. $\vec{\nabla k_{max}}$ is a vector which can be written as $\left(\frac{\partial k_{max}}{\partial x},\frac{\partial k_{max}}{\partial y},\frac{\partial k_{max}}{\partial z}\right)$. This means the rate of changes of maximal principal curvature in x, y and z directions, whereby the calculation of the maximal principal direction $\vec{t_{max}}$ is addressed in [13, 28].

Derivatives of the implicit function are extensively used in the formulae to calculate various related geometry properties that are necessary to compute crest lines on the surface. Unlike the calculations conducted on parametric functions and well-defined explicit mathematical functions, the derivative estimations used in our method take advantages of finite difference methods since the data we work on is discrete in nature. All these geometry properties are calculated based on volumetric data rather than mesh data. A separate study on finite difference error analysis of surface geometry properties [31] finds the central difference approximation is the best finite different method to estimate the geometry properties of a convex surface.

On account of the nature of the implementation of implicit surface polygonizer, we are not able to directly procure all the geometry properties required to assemble crest lines of a surface based on the information from volume data itself. The very element that requires further effort is the gradient of the maximal curvature. To estimate it, we propose a weight sum function based methods by exploring the information based on mesh and experiment the accordingly.

**Fig 10:**
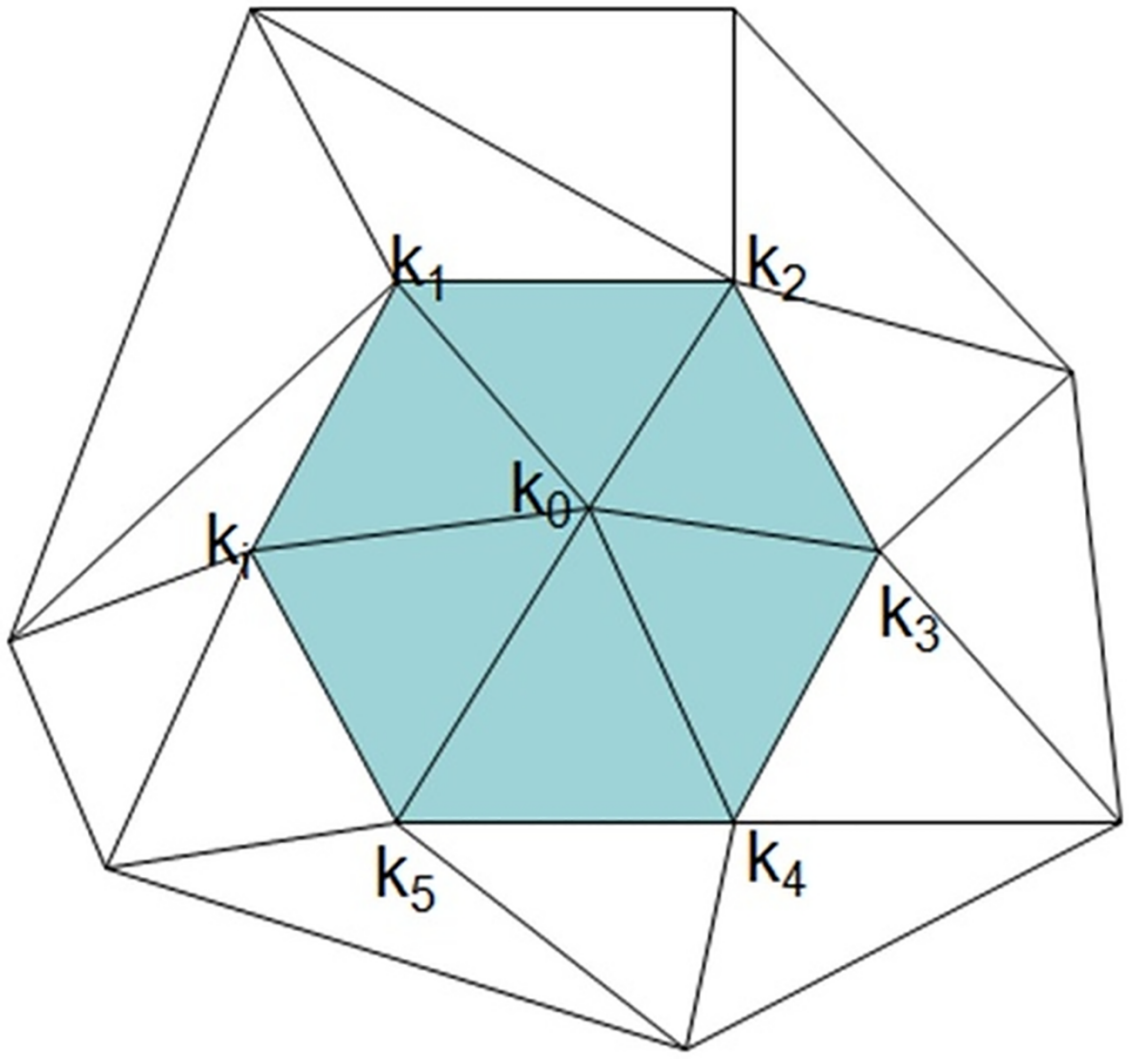
An example of first ring neighbourhood of the vertex of interest in a polygon mesh.

By considering different facts in a calculation, weight function can be used to estimate derivatives in a discrete setting. The weight function takes the influences of the neighbour vertices of a particular vertex of interest to calculate the derivatives of k in (Fig 10). The figure shows a mesh fragment which is the first-ring neighbourhood of the target vertex, i.e. *k*_0_ in this case. The value of principal curvature of all vertices are calculated and known at this stage. The aim is to obtain $\left(\frac{\partial k_{0}}{\partial x},\frac{\partial k_{0}}{\partial y},\frac{\partial k_{0}}{\partial z}\right)$

After extracting the surface, the surface mesh provides us several geometry elements which can be utilized to approximate the gradient of the principal curvature. The geometry elements that are

**Fig 11:**
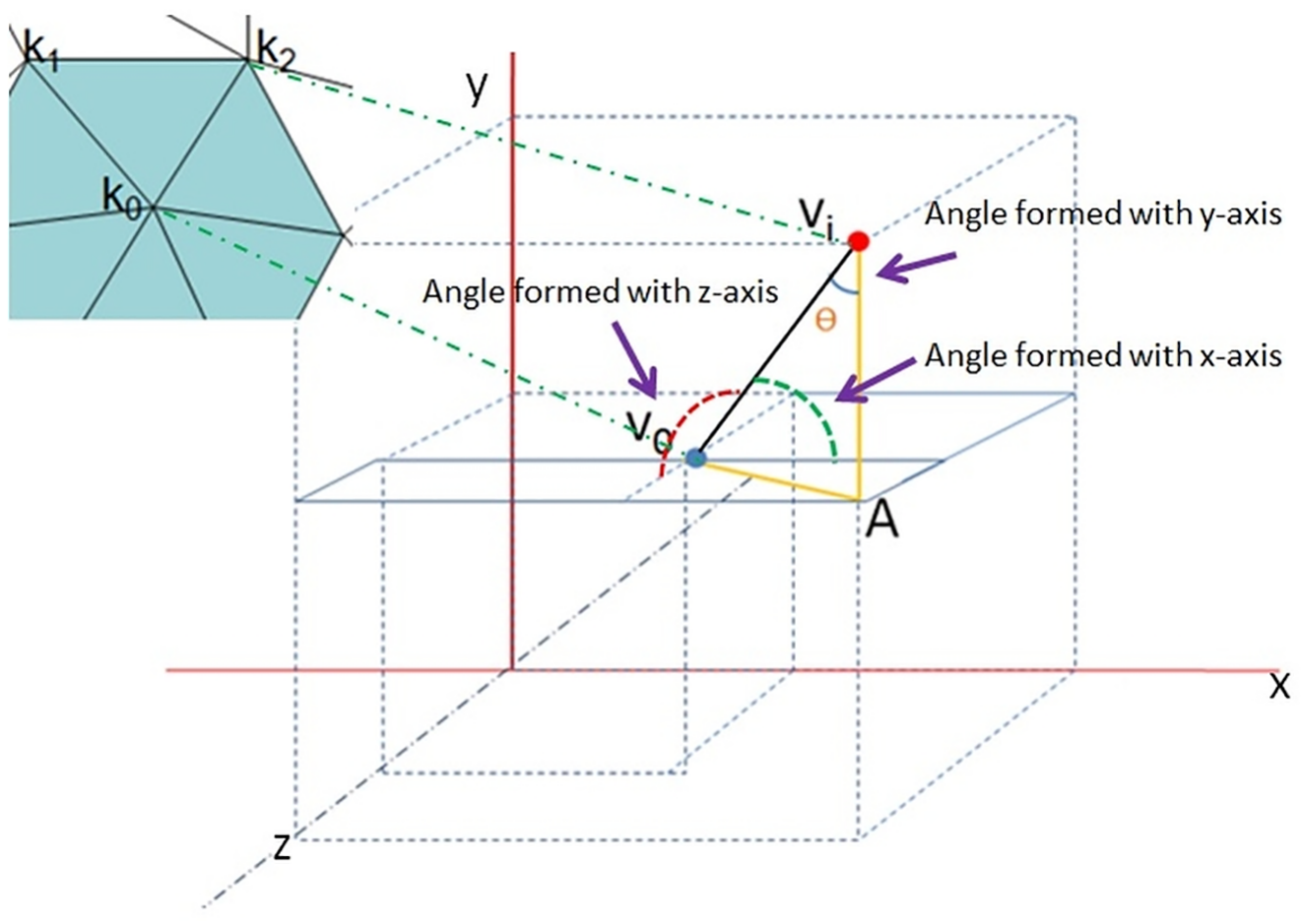
The angle illustration in the weight function.

- Angle: The angle is defined as the corner formed by the edge of the triangle facet to the respective axis (Fig 11).
- Distance: The distance is defined as the interspace reach between the target vertex and the respective neighbour, which means the Euclidian distance.

The assumption is based on the facts that the value at a vertex is influenced mainly by the vertices which are spatially close, i.e. first ring neighbourhood, and have similar intensity. The equation is designed to have the general form (in x-direction) as below

$$\begin{array}{c} \frac{\partial k_{0}}{\partial x}=\frac{\sum_{i=1}^{n}W_{i.x}\left(k_{i}-k_{0}\right)}{\sum_{i=1}^{n}\left|W_{i.x}\right|} \end{array}$$ (2)

where *W*_*i*.*x*_ is the weight function. *k*_0_ is the maximal curvature of the target vertex. *k*_*i*_ is the maximal curvatures of the first-ring neighbourhood. *n* is the total number of the neighbours. The first method of the weight function to approximate the gradient of the maximal curvature considers the angles and the distances. Ergo it is expounded as

$$\begin{array}{c} W_{i.x}=\lambda W_{i.a.x}+\left(1-\lambda \right)W_{i.d.x} \end{array}$$ (3)

λ is the weight control parameter. We believe that the *distance* (*W*_*i*.*d*.*x*_), is inverse proportional to the influence of the neighbour to the target vertex, so is the *angle* (*W*_*i*.*a*.*x*_). Hence, the cotangent of the *angle* is direction proportional to the influence *k*_*i*_ to *k*_0_. So we have

$$\begin{array}{c} W_{i.a.x}=\cot\theta =\frac{\left(x_{i}-x_{0}\right)}{\sqrt{{\left(y_{i}-y_{0}\right)}^{2}+{\left(z_{i}-z_{0}\right)}^{2}}} \end{array}$$ (4)

*x*_0_, *y*_0_ and *z*_0_ are the coordinates of the target vertex and *x*_*i*_, *y*_*i*_ and *z*_*i*_ are the coordinates of the respective neighbour. To calculate the weight function of the distance, we can just employ the general formula for Euclidean distance calculation in 3D space.

$$\begin{array}{c} Dist_{i}=\sqrt{{\left(x_{i}-x_{0}\right)}^{2}+{\left(y_{i}-y_{0}\right)}^{2}+{\left(z_{i}-z_{0}\right)}^{2}} \end{array}$$ (5)

Owing to the fact that the distance is abundantly miniature, we normalized the distance described below

$$\begin{array}{c} W_{i.d.x}=Dist_{norm}^{i}=\frac{Dist_{i}}{\sum_{i=1}^{n}Dist_{i}} \end{array}$$ (6)

The similar calculation applies to $\frac{\partial k_{0}}{\partial y}$ and $\frac{\partial k_{0}}{\partial z}$. The *distance* function remains the same. The *angle* function needs to be altered to accord the respective directions.

**Fig 12:**
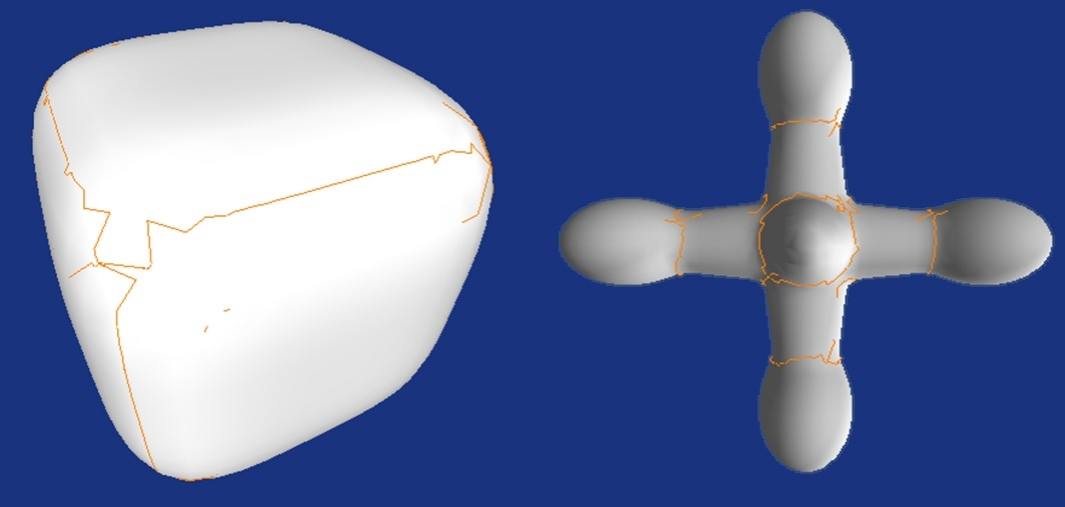
Crest line results on synthetic surface function.

Crest lines extraction results with our methods are shown below (Fig 12).

**Fig 13:**
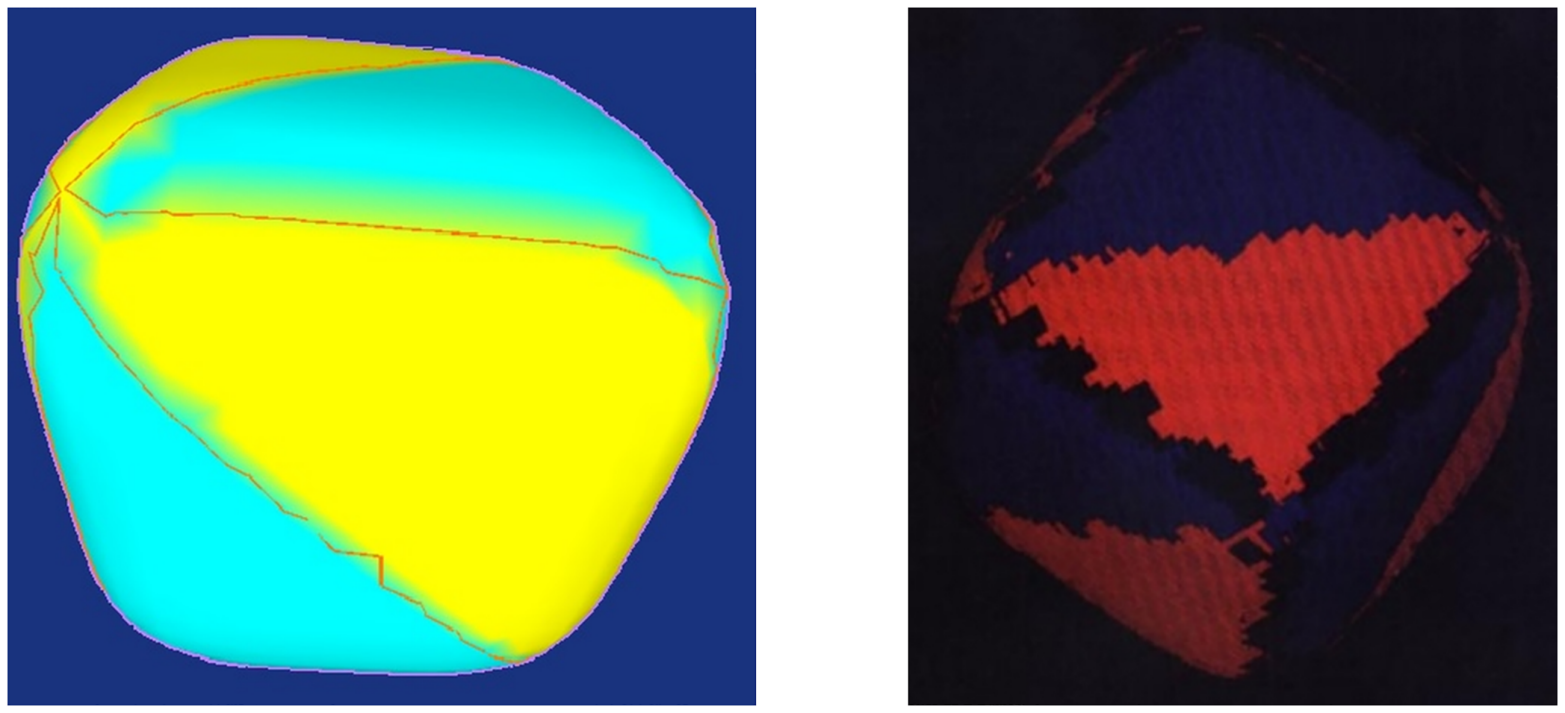
Extremal criterion comparison of crest lines with Monga’s result [28]. **a** Our method. **b** Monga’s results.

Moreover, we highlight all vertices of the surface based on the extremal values in of the optimal result of our work. In Fig 13, the clusters of the vertices in same colour are known as extremal criterions [28].

**Fig 14:**
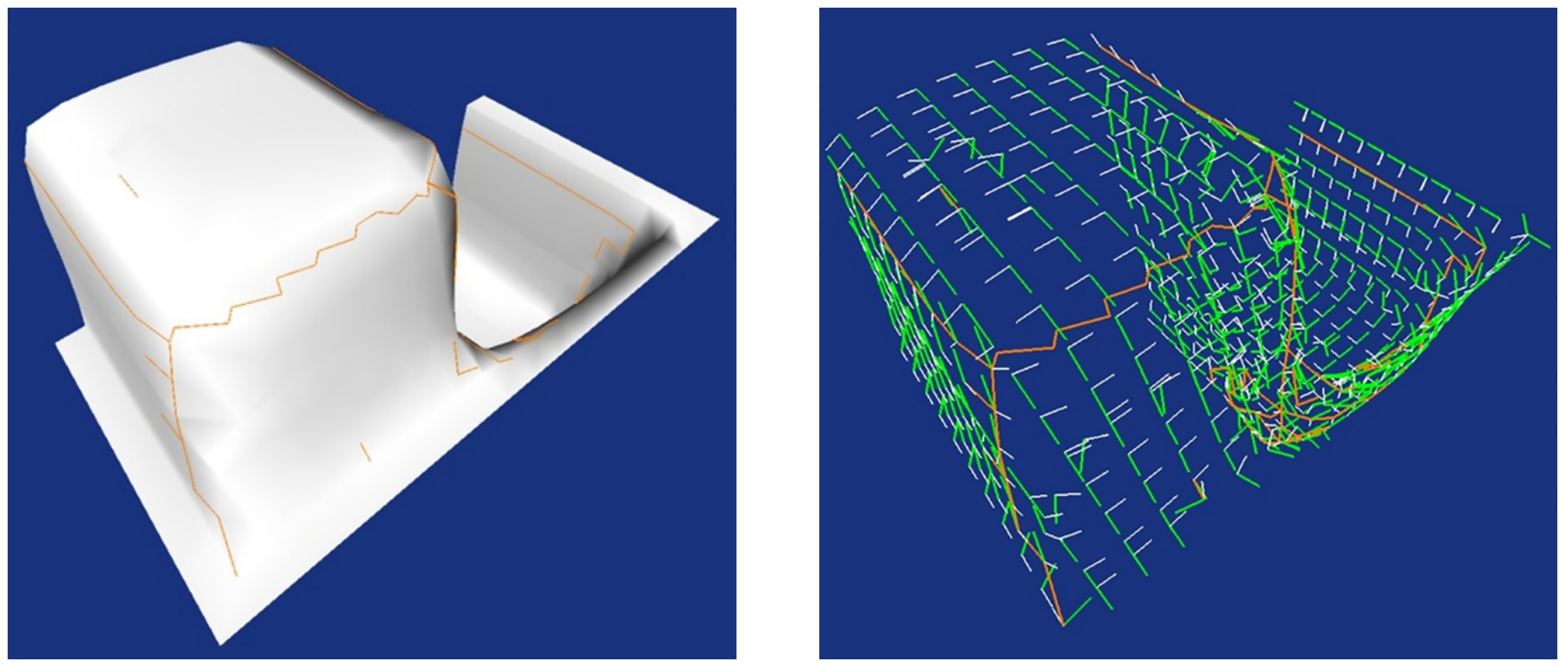
The crest lines extraction results on temperature data. **a** The results overlay the surface. **b** The results overlay principal directions.

We experiment the proposed the method on the real-life engineering and medical data. Fig 14 shows the crest line extraction results on a double glazing window temperature data.The surface represents the same temperature in air.

The results of the proposed crest lines extraction method implemented on synthetic function are reasonably consistent. The results of the method on surface extracted from real-life volumetric data are also sensible yet with some level of inconsistency. There are several notable reasons to be addressed.

- A global scheme: The method developed in this study is a method of global scheme. It is quite challenging to control the behaviour of local details.
- Volume data: The large scale data is obtained from real-life scanning modalities. There are various factors that affect the integrity of the data, i.e. the sampling rate of the scan, the environment noise.
- Numerical concerns: Finite difference methods of numerical analysis are used in the calculation of various geometry properties. The estimations may contain some errors. These errors tend to be accumulated in complex formulae.
- Geometry concerns: Higher order derivatives are usually sensitive to very small variations of a surface. These surface variations may not be visually noticeable by human, yet picked up by the calculations.

## Landmark identification

The third phase of the functional pipeline is the landmark identification on the feature lines. Curvature analysis provides a favourable mean to understanding the nature of a surface. Hence, it is feasible to apply curvature analysis to the feature line extracted to locate landmarks on the surface of an object. Koenderink and Doorn [32] proposed a novel property named curvedness. The curvedness is defined as the root mean square of two principle curvatures

$$\begin{array}{c} C=\sqrt{\frac{k_{1}^{2}+k_{2}^{2}}{2}},C\in \left[0,\infty \right) \end{array}$$ (7)

When the surface is flat, the value of curvedness is zero. When the surface is extremely curved, the value of curvedness approximate to infinity. We use curvedness as the main instrument to identify the most feature intensive points along the crest lines to be our landmarks.

**Fig 15:**
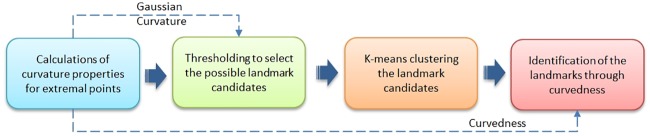
The framework of the landmark identification phase.

Fig 15 illustrates the framework of the final landmark identification phase. It consists of four steps, which are calculations of curvature properties for extremal points, thresholding to select the possible landmark candidates, K-means clustering [33] the landmark candidates and Identification of the landmarks through curvedness.

In step one, the required curvature properties, such as Gaussian curvature and curvedness, are calculated for subsequent steps. This is achieved by interpolating the Gaussian curvatures and the curvedness of the triangle vertices of the surface mesh. Step two uses Gaussian curvature to preliminarily define the potential landmarks by thresholding the extremal points of crest lines. The results of step two are groups of points in the feature intensive regions, which have similar high values of Gaussian curvatures. To characterize the membership of the points, we use K-means clustering method to process the results of step two. To ensure the uniqueness of the landmarks, we select the landmarks by choosing the points with highest curvedness value in the respective clusters.

**Fig 16:**
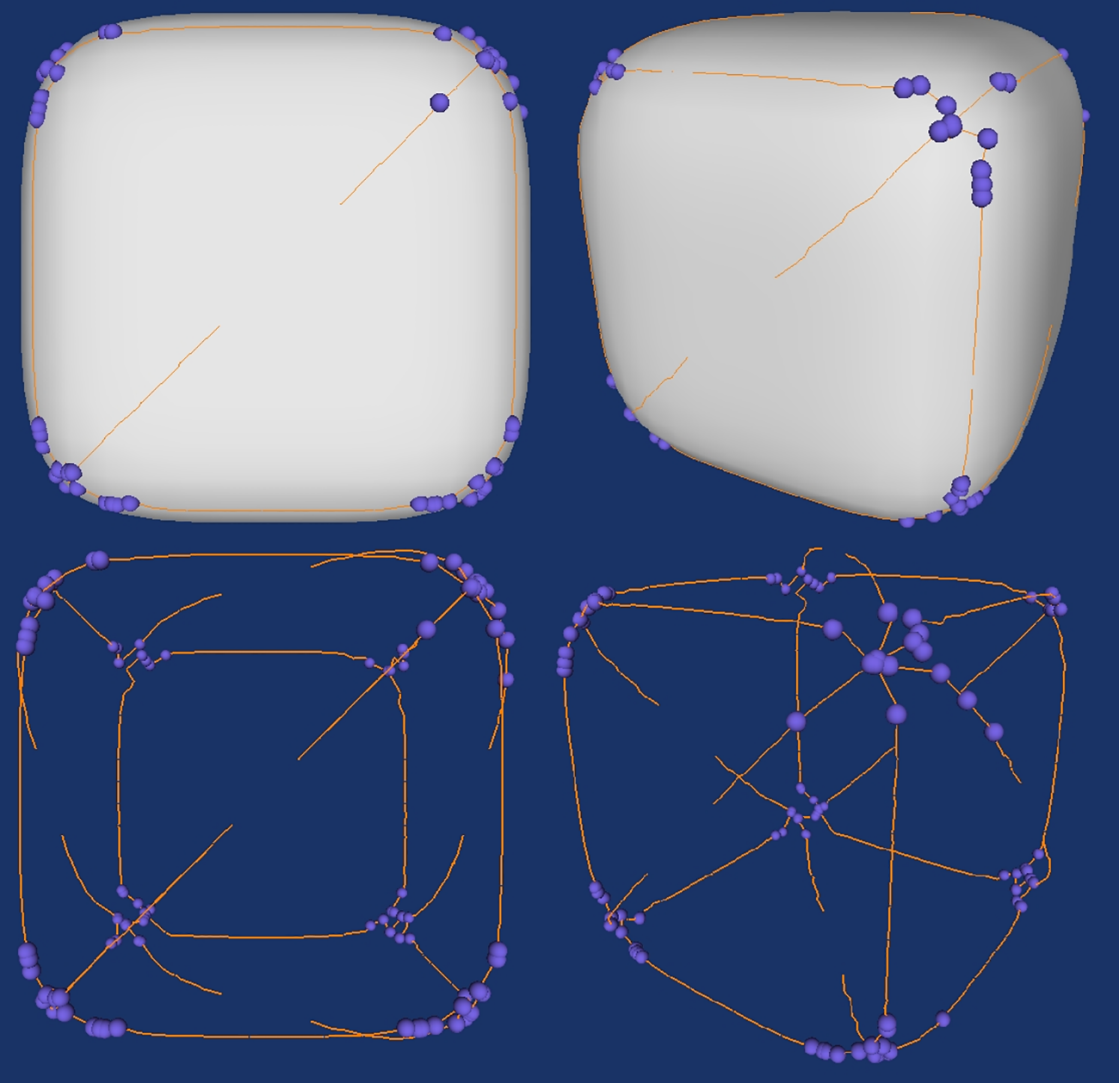
Possible landmarks selected by Gaussian curvature thresholding (upper: The landmark overlaying with the surface and the crest lines; lower: The landmarks overlaying the crest lines).

We first experiment the design of the framework on synthetic data of the smooth cube. Fig 16 shows the result of the step two in the designed framework. In this case, there are 118 points. Although the landmarks are shown in the feature intensive region but they lose the property of uniqueness. We need to select the most distinctive points in these candidate landmarks.

**Fig 17:**
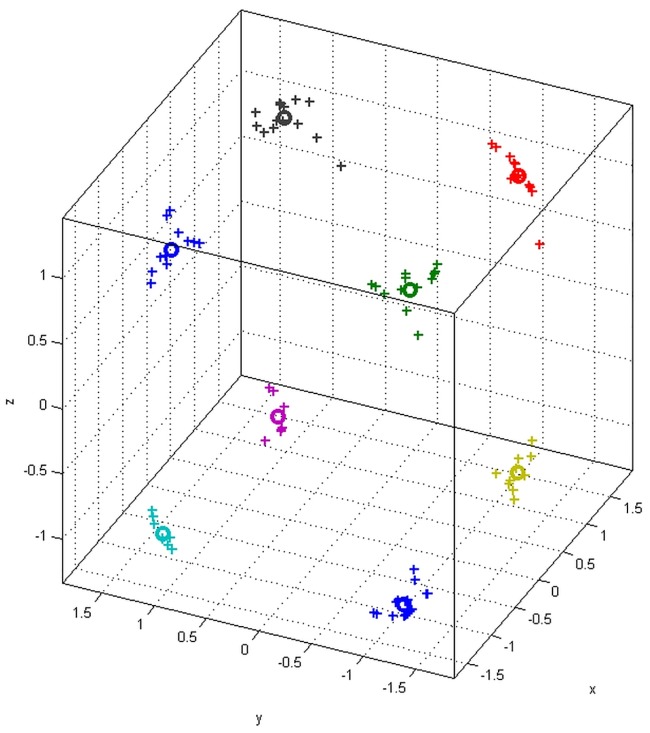
K-means clustering result of the landmark candidates.

Before doing that, we first need to know how many groups/clusters that we can select the landmarks. Fig 17 shows the K-means clustering result of the landmark candidates. K is the number of clusters. The plus sign denotes the points and the circles are the centroids of the clusters. There are eight clusters and the memberships of the points are highlighted in different colours.

**Fig 18:**
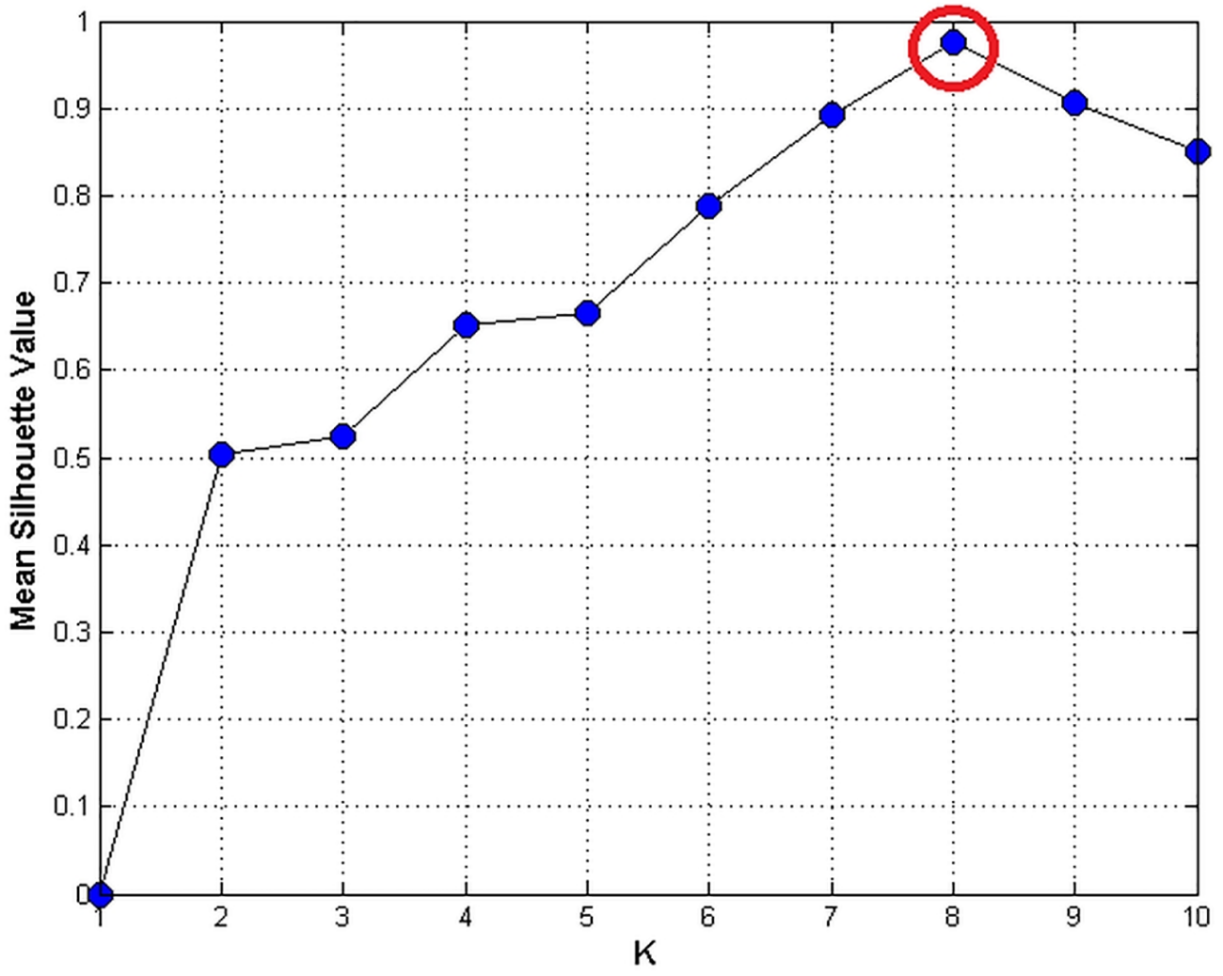
The mean Silhouettes evaluation of the number of clusters obtained by K-means.

K-means clustering is an unsupervised clustering method, but the number of the clusters must be provided prior the clustering process. Heuristically we understand that for the synthetic smooth cube data there are eight clusters (*K* = 8) for the possible landmarks. However, if the surface is defined in a more complex arrangement, then it might not be possible for us to subjectively define the cluster. Rousseeuw [34] proposed a concept, Silhouettes, to evaluate the optimal number of clusters. A higher value of mean Silhouettes coefficient indicates a more optimal clustering result. Fig 18 shows the mean Silhouettes coefficient evaluation of the clusters. The mean Silhouettes coefficient ranges from -1 to 1. Negative value indicates inappropriate clustering. The closer the value approaches to 1, the more optimal the clusters are.

**Fig 19:**
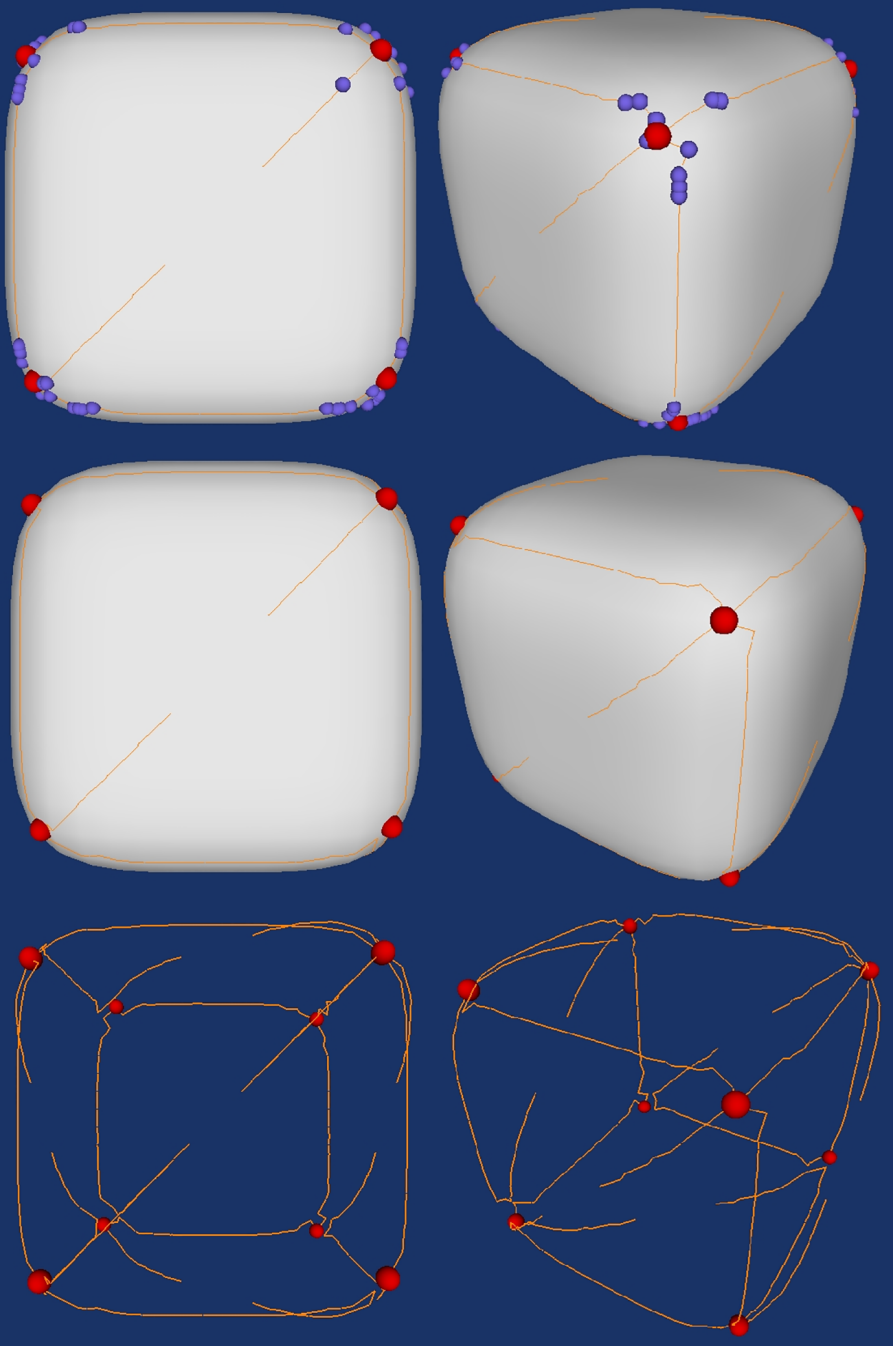
The final landmark identification result on smooth cube data (upper: Landmarks overlay with landmark candidates and the surface; middle: Landmark overlay with the surface; landmarks overlay with the crest lines).

It proclaims that the most optimal number of clusters is eight. The result also coincides with the “rule of thumb” [35] which is a simple way to define the number of clusters for K-means clustering

$$\begin{array}{c} k\approx \sqrt{\frac{n}{2}} \end{array}$$ (8)

where n is the number of data points and k is the approximated number of cluster. In above case, there are 118 data points. The k value calculated by equation above is 7.68 which can be rounded up to 8. After clustering the landmark candidates, we select the points with highest value of curvedness in each cluster. Fig 19 shows the final landmark identification results.

**Fig 20:**
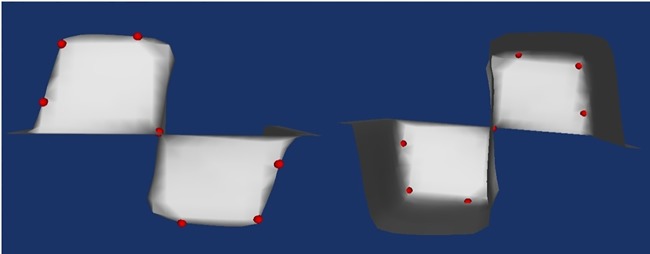
The final landmark identification result on temperature data (left: Front view; right: Back view. Volume size: 10 × 10 × 8; Surface: 633 vertices, 1117 triangles).

After we experiment the method on synthetic data, the method is experimented on real data. We first test it on small size temperature data. The result is shown in Fig 20.

**Fig 21:**
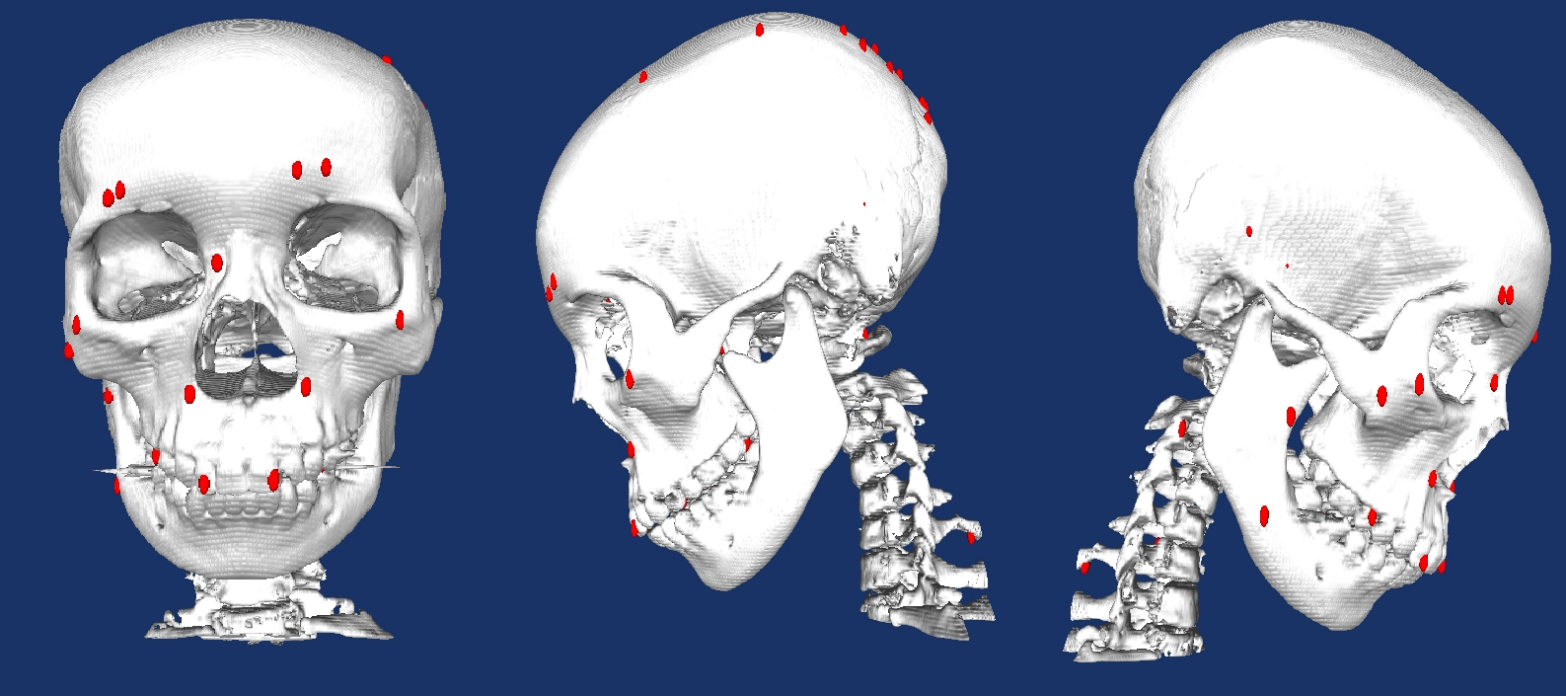
The final landmark identification result on craniofacial data (left: Front view; middle: Left view; left: Right view. Volume size: 512 × 512 × 196;Surface: 1009539 vertices, 2021048 triangles).

Finally we experiment the framework implementation on real pre-processed CT-scanned craniofacial data. The result is shown in Fig 21.

The designed method shows decent results on synthetic data and small scale data. It manifests certain level of inconsistence on huge data. Here are some justifications,

- Huge data inevitably contains some noise, specially for real-life data.
- There are accumulated numerical and other errors are carried from the crest lines extraction phase of the pipeline, which have impact on the result of this phase.
- The number of clusters eventually defines the number of landmarks. For huge size of data, it is difficult for K-means clustering method to achieve a very optimal result.

## Conclusion and future works

As one of the major efforts of this study, a high-level design is conceptually proposed as a functional pipeline which is used to guide the landmark identification process. The pipeline is designed in a modularized way, where each phase of the pipeline is an independent module itself and performs a dedicated function. Each phase works as a black-box that takes input from previous phase and outputs for the next with no influence to each other. This design facilitates the situation when there are additional plug-ins or extensions to the pipeline. The functional pipeline is designed in three-fold. The first phase is the surface extraction phase. We choose the implicit surface polygonizer to be the surface extraction algorithm, which best suits the pipeline in terms of complexity and efficiency. The second phase is the feature line extraction. The feature lines which concern us are crest lines. We provide a weighted function method to calculate the important parameter, gradient of the maximal curvature, of obtaining the crest lines. The ultimate goal is to identify the salient points, i.e. landmarks, which represent the predominant features of a surface. Surface information such as curvature is used to draw the baseline of selecting the landmarks candidates. Eventually we use K-means clustering and curvedness to decide final placement of landmarks.

There are several issues and additional works which can be considered for future work within the functional pipeline design. In the surface extraction phase, the implicit surface polygonizer is an efficient non-exhaustive algorithm, but it suffers the problem of multiple objects surface detection. There are potential works on multiple seed point placements. Multiple seed points can be initiated for multiple objects. As parallel computing becomes the trend. Multiple seed points can utilize the power of parallel processing to achieve an efficient implementation. In crest line extraction and landmark identification phase, there may need a surface smoothing and anti-aliasing process, so more optimal results can be obtained for real-life data. The clustering method used in this study is relevantly conventional. it is potentially use latest spiking neural net model, e.g. spiking neural p system [36, 37], evolutionary algiorithm [38]. In terms of applicatoins, the proposed pipeline framework may be used in 3D tooth feature extraction, human organ feature identification and also motion analysis.
